# Multidisciplinary Management of Skull Metastatic Follicular Thyroid Cancer in a Resource-Limited Setting

**DOI:** 10.1210/jcemcr/luae080

**Published:** 2024-05-27

**Authors:** Yisihak Suga, Mulualem Wondafrash, Metasebia Worku Abebe, Helagenet Eshetu

**Affiliations:** Department of Surgery, Endocrine and Breast Surgery Subunit, Saint Paul's Hospital Millennium Medical College (SPHMMC), PO Box 1271, Addis Ababa, Ethiopia; Department of Neurosurgery, Saint Paul's Hospital Millennium Medical College (SPHMMC), PO Box 1271, Addis Ababa, Ethiopia; Department of Surgery, Plastic and Reconstructive subunit, Saint Paul's Hospital Millennium Medical College (SPHMMC), PO Box 1271, Addis Ababa, Ethiopia; Department of Surgery, Saint Paul's Hospital Millennium Medical College (SPHMMC), PO Box 1271, Addis Ababa, Ethiopia

**Keywords:** multidisciplinary, skull metastasis, follicular cancer, limited resource

## Abstract

A 60-year-old woman presented to the Department of Surgery with an anterior neck mass and a mass on her left forehead. She was diagnosed with follicular thyroid cancer with metastasis to the skull, a rare presentation of follicular thyroid cancer that is associated with a poor prognosis. A multidisciplinary team evaluated the patient and devised a 3-staged surgical management plan: total thyroidectomy with central lymph node dissection, cranial metastasectomy, and cranioplasty with autologous split rib graft. This case illustrates how innovative multidisciplinary surgical management can be applied in a low-resource setting involving 3 surgical sub-specialties for the best possible outcome in a patient with metastatic follicular thyroid cancer.

## Introduction

Skull metastasis from thyroid cancer is extremely rare, accounting for only 2.5% of all bone metastasis [1]. The preferred management strategy in most patients with bone metastatic thyroid cancer of follicular cell origin is surgical resection of all loco regional disease, if possible, followed by 131I therapy for radioactive iodine (RAI)-responsive disease, external beam radiation therapy or other directed treatment modalities such as thermal ablation, and then thyrotropin (TSH)-suppressive thyroid hormone therapy for patients with stable or slowly progressive asymptomatic disease. Systemic therapy with kinase inhibitors (preferably by use of FDA-approved drugs or participation in clinical trials) in patients with progressive disease that is RAI refractory [2].

## Case Presentation

A 60-year-old woman presented with a 2-month history of a left anterior neck mass. It was associated with voice change and a left scalp mass with no associated headache, seizures, change in behavior, weakness, or speech difficulty. On head and neck physical examination, there was a pulsatile, firm, and mobile 5.7-cm mass on the left frontal skull. Additionally, the thyroid was enlarged on the left side and the mass was firm, with no attachment to the skin or underlying pre-tracheal fascia. There were also cervical lymphadenopathies with asymmetric cortex.

## Diagnostic Assessment

**Figure 1.**
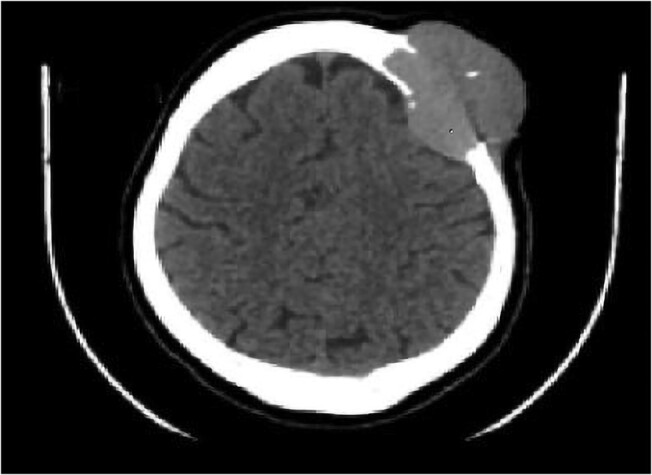
Axial image on MRI showing left frontal bone mass that is well-circumscribed with intra- and extra-cranial component and mass effect on the left frontal lobe.

She had a neck ultrasound that revealed a 4.3-cm left lobe hypo echoic mass with irregular borders and increased vascularity. Additionally, a hyperechoic left upper lobe mass was also seen. Scalp ultrasound showed a 3 × 3.5 cm ill-defined, hyper vascular left frontal lesion that extended into the skull bone with mass effect on the underlying dura. Fine needle aspiration cytology revealed follicular neoplasm both from the thyroid and the skull lesion. Thyroid function tests were within the normal range. Brain magnetic resonance imaging showed a large, spherical, well-circumscribed mass lesion measuring 5.7 × 5.9 × 7 cm in size in the left frontal skull having T1 and T2 predominantly homogenous iso-intense signal to the adjacent muscles. The mass had both an intra- and extra-skeletal component, with associated compression of the underlying left frontal lobe parenchymal but no obvious parenchymal infiltration or adjacent parenchymal signal changes seen. The mass showed diffuse contrast enhancement on post-contrast study, but no significant restriction seen on diffusion weighted imaging (DWI) and apparent diffusion coefficient (ADC) mapping. There was compression and elevation of the overlying scalp tissue (Fig. 1). Other available metastatic workups, such as chest x-ray and abdominal and pelvic ultrasound studies, were negative.

## Treatment

After having multidisciplinary discussion (Endocrine Surgery, Neurosurgery, and Plastic and Reconstructive Surgery), it was decided to do the thyroid surgery first, followed by the skull. Thus, a total thyroidectomy with central neck dissection was performed, and the patient was discharged without complications and put on levothyroxine 200 mcg orally daily.

**Figure 2.**
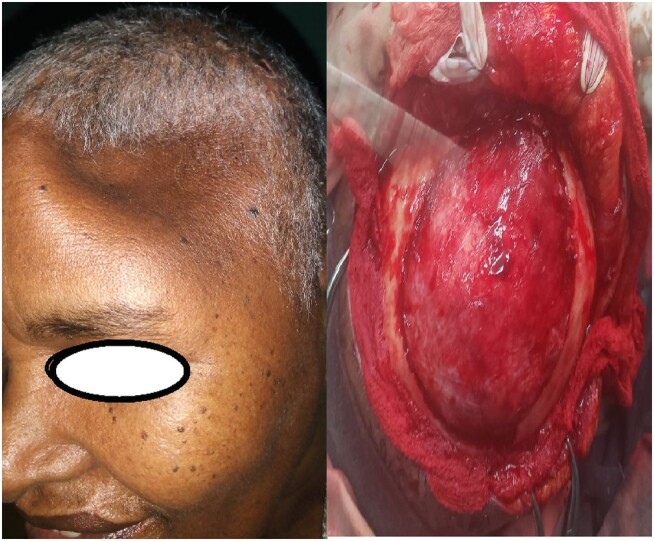
Intraoperative left frontal bone defect after excision of the bone metastasis (left panel) and postoperative skull defect after cranial metastasectomy (right panel).

Two months later, she had the skull lesion resected by neurosurgeons. The intraoperative finding was a 10 × 10 cm left frontal soft, hemispheric mass with erosion of the bone, which was resected with a 1.5 cm margin. The involved dura was also excised and a duraplasty was performed to repair the defect in the dura (Fig. 2). She had an uneventful postoperative course from her craniectomy.

**Figure 3.**
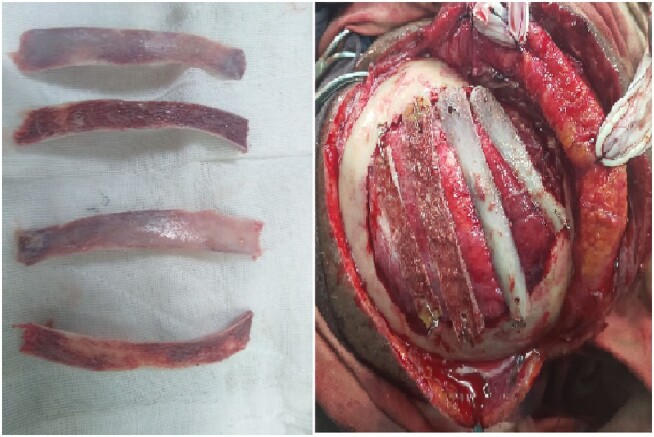
Harvested sixth and seventh rib before and after being placed on the cranial defect.

Three months after the second surgery, she was operated on by the plastic and reconstructive surgeons. The sixth and seventh ribs were harvested from the right side of her chest wall and wired with adjacent cranial bone (Fig. 3) after splitting it into 4 segments to reconstruct the defect. She had an uneventful postoperative course from this surgical procedure as well.

## Outcome and Follow-Up

**Figure 4.**
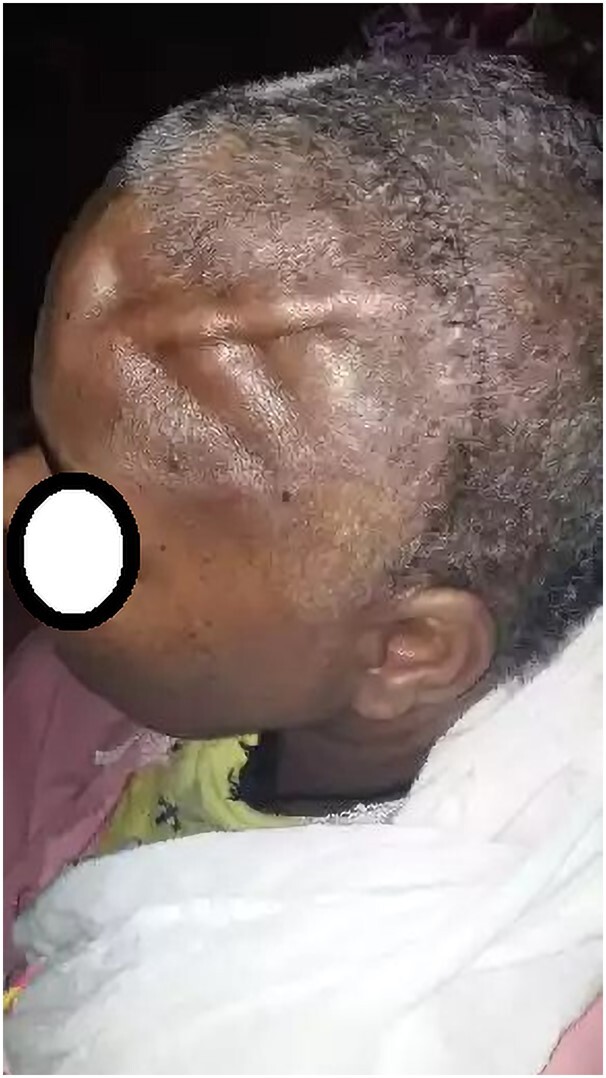
At 1-month postoperative follow-up visit.

Currently, the patient is on her twentieth month after the last surgery and doing well on levothyroxine, adhering to the planned active postoperative surveillance. Her wound has healed, and the depressed part of the skull achieved ideal contour at 1 month following the final surgery. (Figure 4). No signs of recurrence in the thyroid bed, neck, skull, or other body parts were observed during her recent surveillance visit, both in imaging and upon physical examination.

The patient did not receive RAI ablation therapy because it is not available in the country; serum thyroglobulin and antibodies are also not available.

## Discussion

Follicular thyroid cancer is the second most common thyroid cancer after papillary thyroid cancer. Follicular thyroid cancer is common in areas where iodine deficiency is prevalent and in patients with long-standing goiter [2]. It occurs more frequently in women, and usually presents in the fifth and sixth decades of life. In 1% to 9% of patients with follicular thyroid cancer, metastasis to the bone, liver, and lung are present at diagnosis [3]. In patients where metastatic disease was diagnosed at initial presentation, the predominant sites were the bones (spine, pelvis, hip, and scapula) (42%), followed by lungs (33%), brain (17%), and lymph nodes (8%) [4]. A solitary bony metastasis is a rare initial presentation of follicular thyroid cancer, with a skull lesion being particularly rare, accounting for 2.5% of all bone metastasis [1, 5] Calvarial metastasis from thyroid cancer can occur at any age, with a higher rate of occurrence in female patients. Mean duration from initial diagnosis of thyroid cancer to calvarial metastasis is variable, ranging from 4 to 52 years. The most common findings of the metastasis were soft, hemispheric mass on the skull that is highly vascular and causing destruction/osteolysis of the skull bone [6] which was also evident in our patient's case. Due to the incredibly small number of cases, there are no guidelines for the treatment of bony metastasis to the skull from a thyroid primary. The gold standard for the management of metastatic thyroid cancer involves total thyroidectomy. A decision is then made with regard to whether resection of the bony metastasis is in the best interests of the patient, with most patients having radioactive iodine therapy and radiotherapy [6]. Disease-specific survival rates at 5 years have been reported to be between 26% and 39% for patients with metastatic disease. The mean survival time for these patients was 4.5 years [6]. Poor outcome in bone metastasis may be due to lack of effective RAI. In our resource-limited setup, whereby the patient cannot get RAI or access external beam radiation, resection of the bone metastasis was considered, since it had a possibility of improving survival. Therefore, 3-staged surgery with local control of the follicular thyroid cancer, distant metastasectomy, and cranioplasty was done.

Cranioplasty after craniectomy is done to reconstruct a protective physical barrier, create a natural convex contour of the calvarium, and prevent sinking skin flap syndrome [7]. There are also several reports in the literature that describe several advantages of cranioplasty, including, but not limited to, enhancement of cerebral glucose metabolism, improvement of cerebrovascular reserve capacity, postural blood flow regulation, and cerebrospinal fluid circulation [8].

Cranioplasty can be done using autologous skull bone, rib grafts, and synthetic materials such as metals (titanium, tantrum etc.), ceramics (calcium phosphate, hydroxyapatite etc.), and polymers (polymethyl methacrylate [PMMA], polyethylene, poly-ether-ether ketone, poly-ether-ketone-ketone, etc.), bioactive fiber-reinforced composite, and demineralized bone matrix [7, 8]. Our patient had autologous split rib graft for the reconstruction, since no synthetic materials were available due to resource limitations.

The optimal timing of cranioplasty should aim to avoid one of the most serious cranioplasty complications—postoperative infection—which can also be affected by factors such as long operation time, early cranioplasty, older age, and female gender [7]. Currently, early cranioplasty is recommended, since it has shown improved clinical outcomes with regard to neurocognitive improvement [9‐11]. In our case, the reconstruction was done at 3 months following the craniectomy, and the patient was discharged without any complications.

Our case highlights that a multidisciplinary team approach for advanced follicular thyroid cancer, using an innovative approach for reconstruction in our limited-resource setting, can result in a good outcome.

## Learning Points

- Solitary skull metastasis is an extremely rare initial presentation of follicular thyroid carcinoma.
- Metastatic lesions to the skull from follicular carcinoma of the thyroid are usually highly vascularized and cause osteolytic lesions with significant local destruction of the bone.
- Even in the resource-limited setup, a multidisciplinary approach to metastatic follicular thyroid carcinomas offers the best possible outcome for patients.

## Contributors

All authors affirm that the work submitted for publication is original and has not been published other than as an abstract or preprint in any language or format and has not been submitted elsewhere for print or electronic publication consideration. All authors made individual contributions to authorship. Y.S. and H.E. were involved in the initial evaluation, diagnostic and metastatic workup of follicular thyroid cancer, and writing initial draft and submission of the manuscript. M.W. was involved in resection of the skull metastasis. M.W.A. was involved in reconstruction of the skull defect. M.W.A. and M.W. were involved in revision and preparation of the final draft of the manuscript. All the listed authors also finally reviewed and approved the final draft of this manuscript.

## Data Availability

Original data generated and analyzed for this case report are included in this published article.

## Abbreviation

RAI: radioactive iodine
